# Impact of Thermal Processing on the Protein Digestibility of Sardines and Sprats

**DOI:** 10.3390/foods14122096

**Published:** 2025-06-14

**Authors:** Ivo Doskocil, Barbora Lampova, Petr Smid, Aneta Kopeć

**Affiliations:** 1 Department of Microbiology, Nutrition and Dietetics, Faculty of Agrobiology, Food and Natural Resources, Czech University of Life Sciences Prague, Kamycka 129, 165 00 Prague, Czech Republic; lampova@af.czu.cz; 2 Department of Chemistry, Faculty of Agrobiology, Food and Natural Resources, Czech University of Life Sciences Prague, Kamycka 129, 165 00 Prague, Czech Republic; smidp@af.czu.cz; 3 Department of Human Nutrition and Dietetics, Faculty of Food Technology, University of Agriculture in Kraków, Balicka 122, 30-149 Krakow, Poland; aneta.kopec@urk.edu.pl

**Keywords:** culinary treatment, nutrition, protein content, protein digestibility, DIAAS

## Abstract

Fish are a valuable source of high-quality protein and essential nutrients, making them an integral component of a healthy diet. However, protein digestibility, influenced by preparation methods, is a critical factor in assessing nutritional quality. This study aimed to evaluate the impact of various thermal processing methods on the protein digestibility of two commonly consumed small pelagic fish species: sardines (*Sardina pilchardus)* and sprats (*Sprattus sprattus*). Protein digestibility was assessed using two complementary approaches: total protein digestibility and the Digestible Indispensable Amino Acid Score (DIAAS). Fish samples were subjected to different cooking methods, including boiling, steaming, baking, and frying. All thermal treatments enhanced protein digestibility compared to raw fish. Fried samples exhibited the highest total protein digestibility, with sardines reaching 92.4 ± 4.3% and sprats reaching 89.5 ± 4.4%. DIAAS values corroborated these findings, indicating superior protein quality in fried fish. While frying yielded the highest digestibility scores, steaming and boiling provided a favourable balance between improved protein quality and lower potential health risks, with baking achieving comparable results.

## 1. Introduction

Globally, fish meat is an important source of high-quality proteins, unsaturated fatty acids, and other essential nutrients. Owing to the growing awareness of the health benefits of fish and their impact on preventing chronic diseases such as cardiovascular disorders, fish are becoming an increasingly important part of the human diet [1]. In this context, there is increasing interest in small pelagic fish such as sardines (Sardina pilchardus) and sprats (Sprattus sprattus), which represent affordable and nutritionally valuable food sources [2,3]. According to the EU Fish Market report, sardines and sprats are among the most commercially significant small pelagic fish species in Europe. In 2022, European production of sardines exceeded 130,000 tonnes, with sprats reaching approximately 104,000 tonnes. These species are particularly important in countries like Spain, Portugal, and Poland, where they support both local fisheries and export markets. Compared to larger or farmed fish such as salmon or cod, sardines and sprats offer a more sustainable and affordable protein source, with a lower environmental impact due to their short life cycles and efficient feed conversion ratios. Additionally, their small size reduces bioaccumulation of contaminants, making them a safer choice in terms of food safety [4].

Sardines and sprats are particularly valued for their essential amino acids and n-3 polyunsaturated fatty acids contents, especially eicosapentaenoic acid (EPA) and docosahexaenoic acid (DHA). These fatty acids are crucial for maintaining cardiovascular health, supporting brain function, and reducing inflammatory processes in the human body [1,5]. In addition to these beneficial fats, sardines and sprats are significant protein sources, which are important for supporting muscle growth and regeneration. Protein digestibility is a crucial indicator of nutritional value; fish muscle, owing to its simple structure with low connective tissue content, exhibits higher digestibility than terrestrial animals [6]. Myofibrillar proteins such as actin and myosin form the main components of fish muscle, and their structure facilitates access to digestive enzymes, increasing the efficiency of amino acid absorption [7,8]. Additionally, these fish contain significant amounts of fat-soluble vitamins, such as vitamins A and D, and minerals, including calcium, phosphorus, and iron, which are essential for bone, muscle, and immune system health [3,9].

However, fish is rarely consumed raw and is usually subjected to various cooking methods before consumption. Heating is a common method in food processing, and techniques such as boiling, baking, frying, and grilling are used to enhance the flavour of food and extend its shelf life [10]. Heat treatment plays a key role in determining digestibility, which can increase or decrease the availability of the ingested protein depending on the temperature and duration of heating [11]. Several studies have reported high protein digestibility in commonly consumed fish species. According to Usydus et al. [3], the protein digestibility of sardines canned in oil reached 95.0%, while that of sprats canned in oil was 93.0%. Smoked sprats exhibited even higher digestibility values, up to 97.7%. In comparison, smoked trout and salmon had digestibility levels close to 98.7% and 98.2%, respectively. These values confirm that sardines and sprats, alongside other cold- and hot-smoked fish products, are excellent sources of highly digestible proteins. However, the study also demonstrated that intensive heat treatment (e.g., sterilization in canned products) may slightly reduce protein digestibility compared to milder processing methods such as smoking or marination.

At temperatures above 100 °C, excessive heat can induce structural changes in muscle proteins, such as the formation of intermolecular aggregates and cross-links, which reduce the susceptibility of proteins to digestive enzymes. This, in turn, impairs the release of peptides and amino acids, thereby decreasing digestibility [12]. Furthermore, high temperatures promote protein oxidation, a process that significantly impacts digestibility by creating resistance to digestive enzymes [13,14]. Oxidation accelerates with heat and transition metals, resulting in disulfide bonds or other types of cross-links, such as dityrosine or amide bonds, which further hinder protein breakdown during digestion [15]. Traditional thermal processing techniques can also lead to diverse effects on protein digestibility, depending on the specific method applied. For example, moist-heat treatments such as boiling and steaming generally promote protein denaturation, which disrupts tertiary structures and improves the accessibility of cleavage sites for digestive enzymes like pepsin and trypsin [16]. In contrast, high-temperature techniques such as frying may induce the formation of disulfide bonds and protein– lipid oxidation products, which can hinder enzymatic hydrolysis. These interactions are particularly relevant in lipid-rich fish species, where oxidized fat–protein complexes may reduce the digestibility of essential amino acids [17].

In contrast, cooking at lower temperatures can lead to favourable changes in protein structure, such as mild oxidation and denaturation, which enhance protein digestibility. Denaturation, which involves unfolding protein structures, exposes hydrolytic sites to digestive enzymes like pepsin and trypsin, improving protein breakdown [12,18]. This time-dependent process means that proteins can begin to denature even at lower temperatures if subjected to prolonged cooking times. Mild oxidation, resulting from lower-temperature cooking, has been shown to increase the digestibility of muscle proteins by making them more susceptible to hydrolysis by digestive proteases. Overall, lower-temperature cooking promotes partial unfolding and exposure of protein-active sites, improving their accessibility to digestive enzymes, while high temperatures cause irreversible changes that decrease digestibility [15].

Despite the known nutritional benefits of sardines and sprats, data on how common culinary processing methods influence the digestibility of their muscle proteins remain limited. Therefore, this study focused on evaluating how heat treatment affects protein quality and nutritional value in these species. Specifically, we aimed to compare the effects of four common thermal treatments—boiling, steaming, baking, and frying—on the protein digestibility and quality of these two fish species. Protein digestibility was evaluated through two complementary approaches. Total digestibility was determined by comparing the amino acid composition of raw samples to the digested samples following in vitro digestion. In addition, the Digestible Indispensable Amino Acid Score (DIAAS) was used to provide a more comprehensive assessment of protein quality while considering the specific requirements of essential amino acids. The thermal treatments selected in this study—boiling, steaming, baking, and frying—reflect widely used household cooking practices rather than industrial or extreme processing conditions. By applying these methods, we sought to identify which thermal treatment most effectively enhances the digestibility and nutritional quality of sardine and sprat proteins, and whether any differences exist between these two commonly consumed small pelagic fish, with the aim of supporting dietary recommendations and food industry applications.

## 2. Materials and Methods

### 2.1. Samples and Sample Treatment

Sardines and sprats were obtained from Kraków and Koszalin, Poland, from seafood warehouses. The sardines originated in Italy, whereas sprats originated in Poland. All fish were sampled during the same production season and were transported under refrigerated conditions. The average total length of sardines ranged from 12 to 17 cm, and sprats ranged from 10 to 15 cm. The fish were fresh, whole, and ungutted upon delivery, and were processed within 24 h of arrival. The fish were prepared for thermal processing by cleaning and removing all inedible parts, such as fins, heads, and viscera. Thermal treatments were conducted on intact fish fillets, not cut into smaller pieces. The average weight of the processed whole sardines was approximately 40–60 g, and sprats 25–35 g. The detailed nutritional parameters of these samples, including proximate composition, amino acid profile, fatty acid profile, lipophilic vitamins, and selected mineral compounds, were previously analyzed and published by Skoczylas et al. [19].

Thermal treatment involves various cooking methods to achieve consumer acceptance. The fish were cooked in boiling water at 100 °C without salt for 10 min. The steamed fish were steamed for 20 min in a steamer (Tefal, Faucogney-et-La-Mer, France) at 100 °C. Baking was performed in a preheated oven (Amica, Wronki, Poland) at 180 °C for 20 min in a heat-resistant glass baking dish without a cover. Frying was performed in a home-type frying pan with hot processed canola oil at 180 °C for 5 min, as previously reported by Skoczylas et al. [19].

All thermal treatments were conducted on fresh samples, and the duration of each process was determined according to Czarnecka-Skubina [20] and validated using consumer acceptance tests. The selected time–temperature combinations were designed to achieve a similar degree of doneness across all treatments, allowing meaningful comparison. After the thermal processing, the samples were cooled passively at room temperature (approximately 22 °C) for 30 min, uncovered, under ambient air conditions to stop further cooking and ensure uniform conditions before further processing. One portion was used to determine the dry matter content, and the other was freeze-dried using a lyophilizer (Christ Alpha 1–4, Gefriertrocknungsanlagen, Germany). These prepared samples were then stored at −80 °C until analysis.

### 2.2. Nutritional Analysis

Nutritional analyses were conducted in triplicates. The dry matter content was measured by drying samples in an oven (Memmert, Schwabach, Germany) at 103 ± 2 °C until a constant weight was reached (Equation (1)).

Equation (1): Determination of dry mass (DM)

(1) $$DM\left(\%\right)\;=\;\frac{\left(C+wet\;sample\right)-\left(C+dry\;sample\right)}{mass\;of\;the\;wet\;sample}\times 100,$$

where C is the mass of the moisture can.

Nitrogen content was analyzed using the Kjeldahl method (ISO 5983-1:2005) [21] with a Kjeltec 2400 analyzer (FOSS, Hillerød, Denmark). Crude protein content was calculated using a nitrogen-to protein conversion factor of 6.25 (Equation (2)).

Equation (2): Determination of crude protein content using the conversion factor of 6.25

(2) $$Crude\;protein\;content\left(\%\right)\;=\;N\left(\%\right)\times CF,$$

where N is the percentage of nitrogen in the sample, and CF is the conversion factor.

The amino acid analysis was performed using Eurofins Food & Feed Testing, Czech Republic s.r.o., accredited by the Czech Institute for Accreditation (accreditation number: 1546). Tryptophan content was measured using liquid chromatography with a fluorescence detector, whereas other amino acids were quantified using ion chromatography with ultraviolet detection.

### 2.3. Static In Vitro Digestion Model

A simulated in vitro static digestion model was developed based on the methodology described by Brodkorb et al. [22]. Briefly, 5 g of the sample were combined with 5 mL of simulated salivary fluid (containing 15.1 mM KCl, 3.7 mM KH_2_PO_4_, 13.6 mM NaHCO_3_, 0.15 mM MgCl_2_·6H_2_O, 0.06 mM (NH_4_)_2_CO_3_, 1.1 mM HCl, and 1.5 mM CaCl_2_·2H_2_O) (all Lach-Ner, Neratovice, the Czech Republic), 0.5 mL amylase (Sigma-Aldrich Prague, Czech Republic) (with a final concentration of 75 U/mL in the digestate), and distilled water to achieve a final 1:1 volume ratio. The mixture was incubated at 37 °C and pH 7 for 2 min.

Next, the oral bolus was blended with 8.1 mL of simulated gastric fluid (containing 6.9 mM KCl, 0.9 mM KH_2_PO_4_, 25 mM NaHCO3, 47.2 mM NaCl, 0.12 mM MgCl_2_·6H_2_O, 0.5 mM (NH_4_)_2_CO_3_, 15.6 mM HCl, and 0.15 mM CaCl_2_·2H_2_O) (all - Lach-Ner)and 1 mL pepsin (Sigma-Aldrich) (with a final concentration of 2000 U/mL in the digestate). The pH was adjusted to 3 by adding 5 M HCl and distilled water to maintain a 1:1 volume ratio. The samples were then incubated at 37 °C for 120 min.

Subsequently, the gastric chyme was mixed with 11 mL of simulated intestinal fluid (containing 6.8 mM KCl, 0.8 mM KH_2_PO_4_, 85 mM NaHCO_3_, 38.4 mM NaCl, 0.33 mM MgCl_2_·6H_2_O, 8.4 mM HCl, and 0.6 mM CaCl_2_·2H_2_O), 2.5 mL bile (with a final concentration of 10 mM in the digestate), and 5 mL pancreatin (Sigma-Aldrich) (with a trypsin activity of 100 U/mL in the digestate). The pH was adjusted to 7 by adding 1 M NaOH (Lach-ner) and distilled water to maintain a 1:1 volume ratio. The samples were then incubated at 37 °C for 120 min. Digestion was halted by freezing the samples at −80 °C.

### 2.4. Determination of Total Digestibility

The digested samples were centrifuged at 3500× *g* for 10 min to isolate undigested proteins from the digested amino acids. The entire digestion mixture was centrifuged, and only the pellet, containing undigested proteins, was analyzed for amino acid content. The supernatant was discarded. Total digestibility was evaluated by comparing the amino acid content of digested and undigested samples. In vitro protein digestibility was determined by adding the amount of anhydrous amino acids (AA) to the digested and undigested portions, as described in Equation (3).

Equation (3): In vitro protein digestibility

(3) $$Total\;digestibility\left(\%\right)\;=\;\frac{\sum AA\;in\;digested\;samples}{\sum AA\;in\;undigested\;samples}\times 100,$$

where ΣAA is the sum of amino acids.

### 2.5. Digestible Indispensable Amino Acid Score Determination

DIAAS was determined following the methodology outlined by the Food and Agriculture Organization of the United Nations [23]. This method defines the DIAAS value as the lowest calculated percentage of the digestible indispensable amino acid reference ratio (DIAA). The DIAA (Equation (4)) for each amino acid was calculated by dividing the digestible indispensable amino acid (IAA) content (mg) per gram of food protein by milligrams of the same dietary indispensable amino acid per gram of reference protein (amino acid scoring pattern). The amino acid scoring pattern values for the reference protein were taken from the FAO report Dietary Protein Quality Evaluation in Human Nutrition [23].

Equation (4): Calculation of digestible indispensable amino acid (DIAA)

(4) $$DIAA\;=\;\frac{digestible\;IAA\;content\;in\;1\;g\;protein\;of\;food\left(mg\right)}{the\;same\;IAA\;in\;1\;g\;of\;the\;reference\;protein\;\left(mg\right)},$$

where IAA is an indispensable amino acid. DIAAS was calculated by taking the lowest value of the DIAA reference ratio and multiplying it by 100 to express it as a percentage.

The DIAAS was calculated using Equation (5).

Equation (5): Calculation of Digestible Indispensable Amino Acid Score (DIAAS)

(5) $$DIAAS\;=\;lowest\;value\;of\;DIAA\;\times \;100\left(\%\right),$$

where DIAA is a digestible, indispensable amino acid.

### 2.6. Statistical Analysis

The data were analyzed statistically through a two-way analysis of variance (ANOVA), with subsequent post hoc comparisons performed using Scheffe’s method and a significance threshold set at α = 0.05. Statistical analyses were performed using Statistica 13.2 software (StatSoft, Inc., Tulsa, OK, USA). F-values were calculated to evaluate the differences between groups. The results are presented as arithmetic means (×) and standard deviations, calculated from three separate sample sets.

## 3. Results

**Figure 1:**
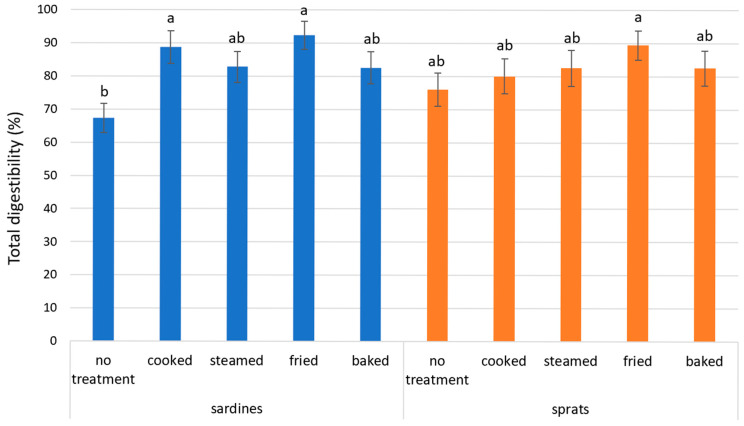
Total in vitro protein digestibility of sardines and sprats. Different lowercase letters are statistically different (*p* ≤ 0.05).

The total in vitro protein digestibility, determined as the ratio of the total amount of amino acids in the dry matter of samples that were not subjected to the in vitro digestion model and digested samples, ranged from 67.4 ± 4.4% to 92.4 ± 4.3% in the analyzed samples (Figure 1). The culinary treatment of the samples was a significant factor influencing the results (*p* < 0.0001; F = 12.10). These results suggest thermal processing significantly improved digestibility, with fried samples showing the highest values. Fried sardines achieved a digestibility of 92.4 ± 4.3%, while fried sprats reached 89.5 ± 4.4%. Although these values showed a trend toward higher digestibility in sardines, the difference between the two species was not statistically significant. The lowest digestibility was observed in the untreated samples. Raw sardines showed a value of 67.4 ± 4.4%, representing a 10% lower digestibility than untreated sprats, which had a value of 76.0 ± 4.9%. Thus, frying increased the total digestibility by 25% for sardines and 13% for sprats. The effect of other culinary treatments, such as baking and steaming, was also positive, although it did not reach the values observed in the fried samples. Baked sardines showed a digestibility of 82.6 ± 4.8%, while baked sprats reached 82.5 ± 5.3%. Similar results were obtained for the steamed samples. Steamed sardines had a total protein digestibility of 82.8 ± 4.7%, and steamed sprats achieved 82.5 ± 5.5%.

When comparing the two fish species across all treatments, sprats generally showed slightly lower total protein digestibility values than sardines, although the differences were not statistically significant. This trend was most evident in the fried samples, where sardines reached 92.4 ± 4.3% and sprats reached 89.5 ± 4.4%. A similar pattern was observed in raw samples, where sardines had the lowest digestibility value of 67.4 ± 4.4%, compared to 76.0 ± 4.9% in sprats. Interestingly, the difference between the two species diminished after baking and steaming, with nearly identical digestibility values (82.6–82.8%). These findings suggest that although both species respond similarly to thermal treatment, sardines may achieve slightly higher digestibility under more intense processing conditions.

The Digestible Indispensable Amino Acid Score (DIAAS) results in Table 1 illustrate that all selected culinary treatments positively affected protein digestibility in fish for both sardines and sprats (*p* < 0.0001; F = 10.78). Fish species had no statistically significant effect on the DIAAS values. The lowest value overall was recorded for raw sardines (85.2 ± 6.2%), followed by raw sprats (98.4 ± 5.6%). For all thermally treated samples, the DIAAS values exceeded 100%. The highest values were observed in fried fish. The overall highest digestibility value obtained by the DIAAS method was for fried sardines (124.4 ± 7.1%). Culinary treatments led to a significant boost in the DIAAS values across all samples. For the thermally treated samples, the DIAAS values ranged from 104.9% to 124.4%, confirming that thermal processing significantly improved the availability of essential amino acids. The highest DIAAS values were observed in fried samples—fried sardines achieved a value of 124.4 ± 7.1%, while fried sprats reached 116.3 ± 6.7%. Steaming and baking also increased DIAAS values, although the effects were less pronounced than those in the fried samples.

Despite the lack of statistically significant differences, sardines consistently achieved slightly higher DIAAS values than sprats across all thermal treatments. This trend was particularly visible in fried and baked samples, suggesting a marginally better amino acid availability in sardines. Nevertheless, both species reached the “excellent protein quality” category (DIAAS > 100%) following any form of thermal processing, confirming their comparable nutritional value when cooked. Additionally, it should be noted that leucine was identified as the limiting amino acid in both species, with sprats showing higher leucine DIAA values in all treatments, which contributed to their higher DIAAS in raw samples. The greater improvement in overall DIAAS observed in sardines after processing likely reflects species-specific differences in protein structure and the extent to which thermal treatment enhances amino acid availability in each fish type.

## 4. Discussion

The digestibility of protein from fish is a key indicator of its nutritional value. It thus plays a crucial role in assessing the quality of fish as an important source of dietary protein, a rich source of high-quality protein with an appropriate profile of amino acids essential for a healthy diet that is gaining popularity. However, the digestibility of fish proteins can be influenced by several factors, such as the type of fish, processing methods, and other nutritional components. Among these factors, the thermal treatment of fish is one of the most significant, as it can dramatically change the availability and usability of proteins in the body [11,24,25].

Our study focused on the digestibility of proteins from sardines and sprats subjected to various culinary treatments, and the results confirmed a significant effect of thermal processing on digestibility. This may be due to protein denaturation, which affects digestive enzyme availability. Changes in the proteins caused by thermal processing can improve their ability to be efficiently broken down and absorbed in the digestive tract. However, culinary processing can reduce protein digestibility under certain circumstances. According to previous studies [26], protein oxidation could potentially contribute to changes in digestibility during thermal processing of fish; however, this was not directly assessed in our study.

In our study, all culinary treatments increased protein digestibility compared to the untreated samples. These findings are consistent with those of Semedo Tavares et al. [11], who investigated the digestibility of proteins in hairtail fillets. Protein digestibility when using pepsin-based digestion was slightly below 60%. When digestion was based on a combination of pepsin and trypsin, higher digestibility was achieved in untreated samples, exceeding 70%, corresponding to the protein digestibility of sprats found in our study. In contrast, the sardines used in the present study exhibited slightly lower digestibility. In a Semedo Tavares et al. [11] study, the highest digestibility, exceeding 90%, was achieved in samples cooked using a combination of pepsin and trypsin. However, the fried fish showed lower digestibility and remained below the 90% threshold. These findings contradict with our results, in which we observed higher digestibility in fried sardine and sprat samples than in cooked samples. The observed difference may be due to different experimental conditions, such as frying temperatures. or heat exposure time. In our study, there was a significant improvement in digestibility during frying, suggesting that this heat treatment may increase protein bioavailability more than heat treatment alone.

Similarly, the effect of thermal processing was also investigated by Jannat Alipour et al. [27], who assessed the impact of frying and grilling on the properties of Persian sturgeon fillets (*Acipenser persicus*). With an initial in vitro digestibility of 81.5% for raw fillets, thermal processing achieved approximately 95% digestibility in grilled and 100% digestibility in fried samples. Although these values are higher than our findings, our conclusions are similar, indicating that fried fish exhibits the best digestibility.

The effect of thermal processing on digestibility was also studied by Jiang et al. [28], who observed changes in the protein quality of the channel fish (*Letalurus punetaus*). Their study showed that the culinary processing of fish significantly improved digestibility compared to raw samples. The highest digestibility values were achieved in steamed samples, where digestibility reached nearly 67%; the roasted samples achieved approximately 63% digestibility; and ordinary cooking resulted in approximately 60%. Although our results demonstrated different levels of digestibility after various treatments, the general conclusion that thermal processing increases the digestibility of fish proteins remains consistent. The difference in the final digestibility values may be due to the varying reactions of different fish species to specific types of thermal processing.

To further contextualize our findings, it is useful to consider how fish species from different aquatic environments respond to thermal treatment. When interpreting the present results, it is important to consider species-related differences in muscle structure and composition that may influence the response to thermal treatment. In addition to muscle protein composition, other factors such as lipid content, connective tissue levels, and individual fish size can also influence the degree of thermal denaturation and subsequent protein digestibility. While we controlled for size variability by selecting fish within a narrow size range, differences in these additional components may partly explain species-specific responses to thermal treatment. Marine species such as sardines and sprats generally contain less connective tissue and higher levels of polyunsaturated fatty acids compared to freshwater species, which may contribute to higher protein digestibility after cooking [6]. In contrast, freshwater fish such as carp or bighead carp may be more prone to oxidative cross-linking and formation of resistant protein aggregates, especially during storage or harsh thermal treatments, leading to reduced digestibility. Liu et al. [29] demonstrated that oxidation of myofibrillar proteins in bighead carp decreased digestibility by promoting aggregation and the loss of essential amino acids, particularly lysine and arginine, which may become inaccessible to digestive enzymes.

Furthermore, fish from different climatic zones exhibit structural adaptations that affect thermal sensitivity. For instance, cold-water species like salmon and trout are more prone to protein denaturation and oxidation at high temperatures, while tropical species such as tilapia show greater thermal stability due to their adaptation to warm environments [6,30]. Despite these interspecies differences, the digestibility of properly cooked fish proteins—whether from marine or freshwater, tropical or frigid origins—remains generally high, with values above 90% reported for most commercially processed products [6]. These findings underline the importance of selecting suitable cooking conditions according to fish type to preserve nutritional quality.

Because traditional measurements of protein digestibility focus solely on the percentage of protein absorbed, this study also assessed digestibility using the DIAAS method. DIAAS provides a broader understanding of protein quality by emphasizing the requirements for essential amino acids. DIAAS values were categorized into three key groups and numerically represented the quality of the protein source based on its essential amino acid content. The overall score, which enables comparisons between different protein sources, reflects the quantity of the limiting amino acid, the essential amino acid present at the lowest concentration in the protein [23,31].

The DIAAS results allowed evaluation of the protein quality of sardines and sprats after various thermal treatments. Fried samples achieved the highest DIAAS values, which fell into the “excellent protein quality” category, with values exceeding 100%. For cooked, baked, and steamed samples, DIAAS values were lower but remained above 100%, placing them in the “excellent protein quality” category. Samples without thermal treatment achieved the lowest values, which were lower than 100% but higher than 75%; therefore, untreated culinary samples can be classified as having “good protein quality.” These findings suggest that sardines and sprats are sources of high-quality proteins, even in raw form, which is essential for dietary habits such as sushi, where fish are often consumed raw. Our results of DIAAS showed that heat treatment significantly improved protein quality in both fish species. This trend confirms that cooking is the traditional method of heat treatment in which nutritional values are preserved. In contrast, frying led to the highest DIAAS values and has an advantage in protein digestibility and protein bioavailability. This advantage may result from a combination of more extensive protein denaturation at higher frying temperatures, improved enzymatic accessibility, minimal leaching of amino acids due to the absence of water, and reduced processing time, which together help preserve amino acid integrity and enhance DIAAS values. This could be important for consumers who prefer quickly prepared meals without worrying about the reduced nutritional quality. However, it should be noted that despite the higher protein digestibility of fried samples, other factors, such as increased fat content and the potential formation of harmful substances during frying, such as trans fatty acids and polycyclic aromatic hydrocarbons, need to be considered. Although frying can increase protein digestibility, it is not a universally healthy option for fish preparation. Choosing an appropriate oil and frying technique is crucial for minimizing the risks associated with this preparation method. Although steaming is generally considered a safe and gentle cooking method, it may lead to some loss of water-soluble vitamins and minerals. Baking, particularly at higher temperatures, can promote the formation of Maillard reaction products; excessive browning may also result in the formation of advanced glycation end-products or polycyclic aromatic hydrocarbons [32,33]. In the long term, consumers should balance higher protein quality with potential risks and prefer healthier preparation methods, such as cooking or baking, which balance nutritional value and health risks [34].

A comparison of our results with the literature shows that, although different studies yield different results depending on the type of fish, processing method, and evaluation method, the general consensus confirms the positive impact of thermal processing on protein digestibility. These findings are consistent with our results, especially for fried samples, where the increase in digestibility was the most significant.

## 5. Conclusions

In conclusion, this study suggests a significant effect of thermal processing on the digestibility and protein quality of sardines and sprats. Thermal treatments such as frying, steaming, and baking substantially improved fish protein digestibility compared to raw samples. The most effective method was frying in canola oil at 180 °C for 5 min, which yielded the highest digestibility and DIAAS values. Steaming was performed at 100 °C for 20 min in a closed steamer, while baking was conducted at 180 °C for 20 min in a preheated oven without cover. These conditions were selected based on established culinary guidelines and validated through consumer acceptance testing. Each of these methods significantly enhanced digestibility, with values exceeding 80% for steaming and baking, and over 90% for frying. Although the positive effects of frying on protein bioavailability have been demonstrated, it should not be overlooked that there are health risks associated with frying, particularly the increased fat content and harmful by-products produced during this heat treatment. Alternative cooking methods like steaming and baking have balanced nutritional value and potential health risks. Overall, sardines and sprats are high-quality protein sources, and their nutritional benefits can be maximized through specific, validated cooking conditions that promote both digestibility and safety.

## Figures and Tables

**Table 1:** Digestible Indispensable Amino Acid Scores (DIAASs) of sardines and sprats.

DIAA a DIAAS										
Fish	Sardines					Sprats				
Culinary Treatment	No Treatment	Cooked	Steamed	Fried	Baked	No Treatment	Cooked	Steamed	Fried	Baked
Histidine	1.17 ± 0.08 ^b^	1.51 ± 0.07 ^ab^	1.44 ± 0.08 ^ab^	1.69 ± 0.10 ^a^	1.48 ± 0.08 ^ab^	1.26 ± 0.07 ^b^	1.32 ± 0.08 ^ab^	1.39 ± 0.08 ^ab^	1.46 ± 0.08 ^ab^	1.36 ± 0.08 ^ab^
Isoleucine	1.16 ± 0.08 ^b^	1.57 ± 0.08 ^a^	1.46 ± 0.08 ^ab^	1.70 ± 0.10 ^a^	1.50 ± 0.09 ^ab^	1.35 ± 0.08 ^ab^	1.41 ± 0.08 ^ab^	1.48 ± 0.08 ^ab^	1.64 ± 0.09 ^a^	1.41 ± 0.08 ^ab^
Leucine	0.85 ± 0.06 ^b^	1.13 ± 0.06 ^ab^	1.07 ± 0.06 ^ab^	1.24 ± 0.07 ^a^	1.11 ± 0.06 ^ab^	0.98 ± 0.06 ^ab^	1.05 ± 0.06 ^ab^	1.11 ± 0.06 ^ab^	1.16 ± 0.07 ^a^	1.07 ± 0.06 ^ab^
Lysine	1.04 ± 0.08 ^c^	1.53 ± 0.07 ^ab^	1.40 ± 0.08 ^abc^	1.66 ± 0.09 ^a^	1.42 ± 0.08 ^ab^	1.26 ± 0.07 ^bc^	1.41 ± 0.08 ^abc^	1.49 ± 0.08 ^ab^	1.51 ± 0.09 ^ab^	1.42 ± 0.08 ^abc^
SAA	1.30 ± 0.06 ^c^	1.63 ± 0.07 ^abc^	1.38 ± 0.08 ^bc^	2.01 ± 0.11 ^a^	1.40 ± 0.08 ^bc^	1.48 ± 0.08 ^bc^	1.66 ± 0.09 ^abc^	1.74 ± 0.10 ^ab^	2.00 ± 0.11 ^a^	1.64 ± 0.09 ^abc^
AAA	1.38 ± 0.10 ^b^	1.86 ± 0.09 ^a^	1.73 ± 0.10 ^ab^	2.03 ± 0.12 ^a^	1.79 ± 0.10 ^ab^	1.58 ± 0.09 ^ab^	1.70 ± 0.10 ^ab^	1.76 ± 0.10 ^ab^	1.92 ± 0.11 ^a^	1.73 ± 0.10 ^ab^
Threonine	1.26 ± 0.09 ^b^	1.71 ± 0.08 ^a^	1.61 ± 0.09 ^ab^	1.83 ± 0.10 ^a^	1.68 ± 0.10 ^ab^	1.46 ± 0.08 ^ab^	1.56 ± 0.09 ^ab^	1.60 ± 0.09 ^ab^	1.70 ± 0.10 ^a^	1.61 ± 0.09 ^ab^
Tryptophan	1.69 ± 0.08 ^d^	2.08 ± 0.07 ^abc^	1.89 ± 0.08 ^cd^	2.45 ± 0.08 ^a^	2.01 ± 0.08 ^bcd^	1.96 ± 0.08 ^bcd^	2.06 ± 0.08 ^abcd^	2.13 ± 0.09 ^abc^	2.35 ± 0.08 ^ab^	2.10 ± 0.09 ^abc^
Valine	0.91 ± 0.07 ^b^	1.24 ± 0.06 ^a^	1.16 ± 0.07 ^ab^	1.33 ± 7.12 ^a^	1.20 ± 0.07 ^ab^	1.09 ± 0.06 ^ab^	1.17 ± 0.07 ^ab^	1.23 ± 0.07 ^a^	1.31 ± 6.65 ^a^	1.18 ± 0.07 ^ab^
DIAAS (%)	85.18 ± 6.23 ^b^	112.89 ± 5.65 ^ab^	107.09 ± 6.12 ^ab^	124.43 ± 7.12 ^a^	111.20 ± 6.35 ^ab^	98.36 ± 5.62 ^ab^	104.95 ± 6.00 ^ab^	110.70 ± 6.33 ^ab^	116.29 ± 6.65 ^a^	106.80 ± 6.10 ^ab^

DIAA: digestible indispensable amino acid reference ratio; DIAAS: Digestible Indispensable Amino Acid Score; SAA: sulphur amino acids, AAA: aromatic amino acids. Values are expressed as mean ± standard deviations (*n* = 3); numbers on the same raw followed by different lowercase letters are statistically different (*p* ≤ 0.05).

## Data Availability

The raw data supporting the conclusions of this article will be made available by the authors on request.
